# Predicting healthcare employees' participation in an office redesign program: Attitudes, norms and behavioral control

**DOI:** 10.1186/1748-5908-3-47

**Published:** 2008-11-02

**Authors:** David C Mohr, Carol VanDeusen Lukas, Mark Meterko

**Affiliations:** 1 Center for Organization, Leadership and Management Research, Department of Veterans Affairs, Boston, Massachusetts, USA; 2 Department of Health Policy and Management, Boston University School of Public Health, Boston, Massachusetts, USA

## Abstract

**Background:**

The study examined the extent to which components based on a modified version of the theory of planned behavior explained employee participation in a new clinical office program designed to reduce patient waiting times in primary care clinics.

**Methods:**

We regressed extent of employee participation on attitudes about the program, group norms, and perceived behavioral control along with individual and clinic characteristics using a hierarchical linear mixed model.

**Results:**

Perceived group norms were one of the best predictors of employee participation. Attitudes about the program were also significant, but to a lesser degree. Behavioral control, however, was not a significant predictor. Respondents with at least one year of clinic tenure, or who were team leaders, first line supervisor, or managers had greater participation rates. Analysis at the clinic level indicated clinics with scores in the highest quartile clinic scores on group norms, attitudes, and behavioral control scores were significantly higher on levels of overall participation than clinics in the lowest quartile.

**Conclusion:**

Findings suggest that establishing strong norms and values may influence employee participation in a change program in a group setting. Supervisory level was also significant with greater responsibility being associated with greater participation.

## Background

In healthcare, organizations continually strive to implement new programs designed to improve patient care. Some recent programs have focused on implementing electronic health records [1], pay-for-performance [2], and redesigning practice to meet the needs of chronically ill patients [3]. Several studies have also examined the extent to which different practices may lead to effective implementation of changes, and these studies often focus on beliefs and attitudes of the individual participant [4,5]. Less frequently studied is the impact of the activities of coworkers on participation of their fellow employees on overall implementation success. The purpose of this study was to examine how employee attitudes, perceived group norms, and perceived behavioral control predict participation in an innovative outpatient clinic redesign program designed to improve clinic operations and reduce patient waiting times. To do so we apply a model using variables based upon the Theory of Planned Behavior (TPB) [6,7].

### Open Access

The setting for the study is the U.S. Veterans Health Administration (VA); which operates the largest healthcare organization in the public sector. VA has experienced a surge in demand for care from an aging veteran population requiring more healthcare services [8]. Thus, reducing wait times is a high priority. Beginning in 1999, VA collaborated with the Institute for Healthcare Improvement (IHI) to launch a national initiative to diffuse the principles of Open Access across primary care clinics and five specialty clinics [9]. IHI worked with VA for 18 months to implement Open Access using a Framework for Spread model [10].

Open Access, or Advanced Clinic Access (ACA) as it is called in VA, is a patient-centered approach with the goal of providing clinic appointments at the time the patients wants to be seen [11-13]. The program espouses a set of ten change principles for clinic management based on the concepts of shaping the demand (*e.g.*, working down the backlog), matching supply (*e.g.*, providers have an appropriate number of patients on their panel) and redesigning the system to improve supply and demand balance (*e.g.*, planning for periods of provider absence) [11]. ACA was not a mandated program within VA during the study period, but was strongly encouraged and promoted as a way to alleviate the appointment wait time problem brought on by growing patient demand.

Studies of ACA suggest that it has had a positive impact on several dimensions, including an increase in the proportion of patients being able to see their own provider [12], more productive patient-provider visits, increased physician compensation, higher net gains for clinics, more efficient operations, decreased use of urgent care and higher patient satisfaction scores [13,14], reduced demand for office visits [15], greater sustained enrollment in managed care plans [11], and less work demands on employees [14]. During the first 18 months of the ACA implementation program in 1999, the average waiting time for primary care appointments were reduced from 60 days to 28 days. Furthermore, the improvements were sustained, with waiting times averaging less than 25 days in key clinics in September 2004 [10].

The spread plan involved four components: 1) organizational infrastructure, involving a focus on leadership commitment and support, technical support, measurement system to monitor and provide feedback, knowledge management system to document information, progress, issues, and questions; 2) information, including the distributing information about ACA, making the business case for ACA and transition materials; 3) communication of general and technical information about ACA by identifying and using key respected messengers; and 4) leveraging the dynamics of social systems to promote spread, including the recruitment of individuals who adopt new systems, developing communities of practices, and using various motivators, and incentives.

The current study is part of a larger comprehensive evaluation of the implementation and effectiveness of ACA that examined program implementation structure and activities, staff capabilities, waiting times, and patient satisfaction [16]. A recent article, based on part of the evaluation study conceptual model, presents facility level findings on the factors contributing to implementation of the program from the perspective of employees and facility level program managers [17]. We have also documented the effects of the program on facility level waiting times and patient satisfaction [18]. The present study is focused on examining the extent to which individual attitudes and beliefs were related to participation in specific change program aims. We apply a well-used theory from psychology to guide our study.

### Theory of Planned Behavior

The theory of planned behavior (TPB) [6,7] postulates that behavior is influenced by intentions to perform a specific volitional behavior. Behavioral intentions are influenced by attitudes about the behavior, norms and perceived behavioral control. 'Attitude about the behavior' is a function of beliefs about consequences of the behavior and an evaluation of those consequences. The 'group norms' component is a function of social expectations to perform the behavior and motivation to comply with the social group's wishes. Third, 'perceived behavioral control' is the perception of the ease or difficulty of performing the behavior based on the potential actor's sense of his/her ability and experience.

A review of studies on physical activity has found strong support for this model as predictive of behavior [19]. Further, research conducted in healthcare settings has found positive relationship between physician's attitudes, beliefs, and perceived behavioral control and their promotion of mammography screening [20], education about sexually transmitted diseases provided to adolescents [21], smoking cessation [22], and individual health screening and physician visits [23]. The theory has also been applied in the study of employee turnover [24] among other areas.

However, we were unable to identify any studies of TPB that considered behavior within the explicit context of a team setting. In a team, several individuals are working collaboratively toward a common goals such as the implementation of an innovation. The decision of any given individual to participate in the innovation, and to do so with enthusiasm or just at the margin, may be influenced by the participation and behaviors of coworkers.

For our study, we borrow themes from the model to develop our analysis. We note this is not a pure empirical test of the theory. Instead, we use the theory to help in our testing and identification of factors that may lead to employee participation. We do not test intention to engage in the behavior as specified in the model, but do model the actual behavior itself.

### Current study

We hypothesized that the three components of TPB – attitudes about the behavior, perceived group norms, and perceived behavioral control – would relate positively to extent of behavioral participation in the ACA program. The study was conducted as a cross-sectional analysis. We modeled participation at an individual level and at a clinic level.

## Methods

### Setting

The present study involved the secondary analysis of data from a national evaluation of the ACA program involving 78 medical centers selected to represent a range of average patient wait times [16]. Some of these medical centers had multiple primary care outpatient clinics in different locations. Thus, a total of 92 unique primary care clinics were involved in the study. The overall ACA evaluation study also involved specialty clinics in five areas. However, the practice patterns (*e.g.*, time to see patients, procedures, staffing) of specialty clinics are different on several dimensions (*e.g.*, time to see patients, procedures, staffing) compared to primary care clinics. We elected to focus on respondents who self-described themselves as working in primary care in order to control the scope of the present study.

### Procedure

The data source for the study was a survey of employees working in outpatient care. The survey consisted of four sections. In the first section, 'General Background', employees were asked to indicate the clinical area(s) in which they worked, the amount of time spent in each area, their supervisory status, professional role, and job tenure. In the second section, 'Changes to Improve Clinic Access', employees were asked to indicate their involvement in activities related to each of the ACA key changes, and to report their participation in various ACA spread activities. The third section of the questionnaire focused on issues such as attitudes about the program, perceived group norms, and influences on participating in the program. In the final section, 'Summing Up', employees were asked for their perceptions of the impact of ACA on their own work life, on the quality of patient care, and on patients' satisfaction with care and service.

A point of contact (POC) had been designated at each medical facility as part of the national ACA initiative. Survey packets consisting of a cover letter, questionnaire, and pre-paid business reply envelop addressed directly to a third-party data entry vendor were mailed to each POC, who then distributed the packets to clinic employees. The survey was anonymous; packets were addressed to 'Primary Care Staff Member', and no individual respondent ID number appeared on the questionnaire. To increase response rate, a second wave of surveys was distributed to all employees three weeks later after the initial distribution.

### Participants

A total of 9,053 surveys were distributed to all staff, including physicians, nurses, and program support assistants. The surveys were distributed to five specialty care clinical areas (cardiology, audiology, eye care, orthopedics, and urology) at the 78 evaluation study hospitals, but primary care clinic employees were the focus of the study. We obtained a response rate of 39% overall and 40% in primary care. We excluded respondents (n = 48) who indicated that they spent less than half of their time in primary care.

### Participation in ACA

Participants were instructed to indicate whether or not they directly participated in each of the ten change activities designed to improve access. The ten items of the change initiative were presented along with a brief definition and example using language that would be familiar to employees according to pre-administration review by an advisory group of field staff. For example, the change principle of 'work down the backlog' was clarified by adding the example: 'adding extra overbook slots to schedules, extending clinic hours, adding clinic sessions, reviewing wait list to see if medical needs could be met by phone call or other means.' Responses were coded as either participated (1) or did not participate (0). The total number of activities that the individual participated in was computed. Cronbach's alpha for this measure of extent of participation in the intervention implementation was 0.82. Table 1 displays the list of activities, how they were described, and reported rate of involvement.

**Table 1:** Ten key principles of ACA as assessed on the survey.

**Item**	**Percent of Employees Reporting Involvement**
**Work down the backlog **(for example, by adding extra overbook slots to schedules, extending clinic hours, adding clinic sessions, reviewing wait list to see if medical needs could be met by phone call or other means)	53
**Reduce demand **(for example, by extending reappointment intervals, creating alternatives to face-to-face visits, and using referral guidelines)	51
**Understand supply and demand **(for example, by knowing how many appointment slots a clinic has, knowing what the provider panel size cap is, knowing how many patients come in, call in, or are scheduled each day for the clinic)	52
**Reduce appointment types **(for example, by reducing the number of separate clinic profiles, standardizing the length of appointments)	27
**Plan for contingencies **(for example, by anticipating and planning for situations like provider leaves and the annual flu vaccination season)	40
**Manage the constraint **(for example, by figuring out where the 'logjams' occur in your patient care process and figuring out actions to deal with them)	29
**Optimize the care team **(for example, by using standard protocols, matching patient needs to skills of appropriate team members, not necessarily always a physician)	46
**Synchronize patient, provider and information **(for example, by starting clinic on time, checking charts for completeness, accuracy and presence at appointment)	51
**Predict and anticipate patient needs at the time of the appointment **(for example, by using regular clinic team 'huddles' to communicate and deal with possible situations that may arise, using clinical reminders to get as much done in each visit as possible)	47
**Optimize rooms and equipment **(for example, by having the same supplies available in each exam room, making sure supplies are continuously stocked, using 'open' rooming)	48

* A total of 2,242 employees provided responses. The question stem appears below. Which if any of these efforts to improve veterans' access to care have **you been directly involved in**? *You may check more than one. You should check all changes that you were involved in regardless of whether the change was successful or not*.

### Predictor variables

Three scale scores representing attitude about the behavior, perceived group norms, and perceived behavioral control were computed; items were assigned to scales based on face-validity. All items were assessed on a five-point Likert scale ranging from one (Strongly Disagree) to five (Strongly Agree). 'Attitude about the behavior' was assessed by four items that elicited employee perceptions of the effectiveness of the ACA program, belief that wait times are a problem to be addressed, and whether the program led to improved quality of care. 'Perceived group norms' was assessed with four items. These items elicited employee perceptions regarding leadership priority for the ACA program, the extent of discussion of the program at employee meetings, the extent of team consensus that ACA is being implemented, and that a well-respected team member is vocalizing support for the efforts. 'Perceived behavioral control' was assessed with three items that asked if the team was able to adapt ACA to meet their clinic needs, and about the extent of influence in managing care and making improvements using the model. Cronbach's alpha for the three scales was 0.78, 0.81, and 0.80, respectively.

### Individual and facility control variables

In the model we also included several control variables at both the individual and facility level that prior research suggested might have an effect individuals' participation in the ACA program.

### Individual characteristics

We included variables relating to the individual's role in the clinic: full-time status, clinic tenure, managerial level, and occupation. Individuals were dichotomously coded as having full-time status if they reported at least 32 hours or more per week in the clinic. Clinic tenure was as a dichotomous variable representing whether or not the individual had worked in the same clinic for at least one year. Three dichotomous variables were created to represent level of managerial responsibility. These variables were 'team leader', 'first line supervisor', and 'manager', with individuals having no managerial status as the referent group. We also created dichotomous flag variables for multiple professions, including physician, nurse practitioner, registered nurse, and program support assistant, the referent group in the series were all other clinic employees (*e.g.*, licensed practical nurses, other technical occupations). Finally, we also measured overall job satisfaction using the item: 'Considering everything, how satisfied are you with your job?'. Responses ranged from one (Strongly Disagree) to five (Strongly Agree). Individuals with greater job satisfaction have been found to be more likely to participate in the change programs [27][28].

### Facility context

Two facility level variables were also included in the model: clinic size and teaching affiliation. Clinic size was measured as the number of full-time equivalent employees and was reported by the POC at each facility. This was included in the model because research suggests that size has a pervasive influence on several outcome variables, with larger size having a negative influence on outcomes. We also included teaching affiliation. Resident physicians can work in primary care clinics at teaching affiliated hospitals. Although, residents were not included in our survey there may be differences in the way clinics are operated in academic affiliated settings.

### Data Analysis

The study hypotheses were tested using a hierarchical mixed linear model. We used this approach to account for clustering of employees in primary care clinics. This provides an advantage over traditional ordinary least squares (OLS) models because the interdependence of variables within a given unit of observation is not considered using OLS, and estimates may be misstated. Two models were estimated. The first model excluded employee ratings of attitudes, norms, and perceived behavioral control. We regressed individual characteristics and facility context variables on our computed participation in ACA. In the second model, we included the measures of the three key TPB variables: respondents' attitude toward the intervention, their perceptions of group norms regarding the value of the intervention, and their perceive control over their participatory behavior. By estimating two separate models in this manner we were able to examine the increase in variance accounted for when the predictor variables of interest were added to the baseline model. In both models, clinic was included as a random effects variable to account for the clustering of respondents by clinic.

Missing values were deleted list-wise, so that all observations had complete data for the analysis. Due to missing values, our total sample size was 2,201. We used Optimal Design Software to compute power analysis for our regression model and found our study had power greater than 0.80 to detect small effect sizes of 0.20 with an average of 25 respondents per group and 82 groups [25].

In order to examine how level of participation was affected at the clinic level by our predictor variables, we conducted a second set of analyses. To begin, we created three groups of clinics (top 25%; middle 50%; and bottom 25%) based on their average score for each of the three TPB factors. We then used analysis of covariance (ANCOVA) to compare these groups on their mean level of clinic participation. The covariates included clinic size and teaching affiliation. To provide more stable estimates at the clinic level, we only included those clinics (n = 82) with ten or more respondents.

## Results

### Sample characteristics

The majority of the sample reported working full-time in the clinic (84%) and having been in the clinic for at least one year or longer (85%). Most of the sample (74%) reported not having any managerial authority, 16% indicated they were team leaders, 5% reported being a first line supervisor, and 5% reported being a manager. Regarding profession, 20% of the sample reported being a medical officer, 12% were nurse practitioners, 20% were nurses, and 21% reported themselves as program support assistants. Although there was some variation at the clinic level in the study sample, these percentages approximate the known staffing distributions in VA primary care clinics on a nationwide basis.

The individual participation scale mean was 4.65 (SD = 3.05) on a ten-point scale, indicating that the average respondents engaged in just under half of the ACA change activities. Attitudes about practice, perceived group norms and perceived behavioral control all correlated significantly with participation rate, with correlations ranging from r = 0.24 to 0.32, p < 0.001. We examined the normality of these variables before running regression models; in all cases, the variables were normally distributed with skewness and kurtosis not larger than |1|.

Next, we conducted hierarchical linear mixed model analyses with participation as the dependent variable and clinic as the random effect. All variables were standardized to facilitate comparison of coefficients. Computation of the intraclass correlation indicated that approximately 4% of the variance in staff participation was accounted for at the clinic level [26]. This variance would have been considered as error variance in an OLS model not taking into account the nesting of employees in clinics.

Model 1, including only individual characteristics and facility context, accounted for 17% of the variance based on the pseudo r-square. When we added the three TPB variables (Model 2) the variance accounted for increased to 23%. Of the TPB factors, perceived group norms had the strongest association with participation (β = 0.23, p < 0.01), while attitude about the behavior (β = 0.07, p < 0.01) was less strongly associated, but still significant. Perceived behavioral control, however, was not significant.

Several covariates were significant as well in Model 2: clinic tenure of at least one year (β = 0.15, p < 0.01); being a physician (β = 0.05, p < 0.05); being a nurse practitioner (β = 0.12, p < 0.01); and being a registered nurse (β = 0.09, p < 0.01). The three variables coded to indicate supervisory level were all significant compared to individuals without supervisory status (referent group), further, parameter estimates increased as one moved up the level of supervisory hierarchy: team leader (β = 0.13, p < 0.01); first line supervisor (β = 0.14, p < 0.01) and manager (β = 0.21, p < 0.01).

Non-significant predictors included several individual characteristics (full-time status, job satisfaction, and being a program support assistant) as well as team size and academic affiliation. Descriptive statistics and standardized coefficient estimates from Model 1 and Model 2 for all variables are reported in Table 2.

**Table 2:** Hierarchical Linear Mixed Model Regression Predicting Participation.

			Model 1		Model 2	
	Mean	SD	β	95% CI	β	95% CI

Intercept	--	--	.05	.00–.11	.05	.00–.11
Full-time status (1 = yes)	0.84	--	.01	-.03–.06	.02	-.02–.06
Clinic tenure greater than one year	0.85	--	.14**	.10–.18	.15**	.11–.19
Team leader (ref = no)	0.16	--	.14**	.10–.18	.13**	.09–.17
First line supervisor (ref = no)	0.05	--	.15**	.11–.19	.14**	.11–.18
Manager (ref = no)	0.06	--	.24**	.20–.27	.21**	.17–.25
Physician	0.21	--	.05*	.00–.10	.05*	.00–.09
Nurse Practitioner	0.12	--	.12**	.07–.16	.12**	.07–.16
Registered Nurse	0.20	--	.08**	.03–.13	.09**	.04–.14
Program Support Assistant	0.20	--	-.04	-.09-.01	-.03	-.07-.02
Job satisfaction	3.79	1.22	.18**	.14–.22	.02	-.02–.07
Size of Primary Care clinic	77.45	45.67	.01	-.06–.08	.00	-.07-.06
Academic Affiliation (1 = yes)	0.49	--	-.02	-.09-.04	-.01	-.08-.04
**Step 2**						
Attitudes about ACA	3.47	0.69			.07**	.02–.12
Perceived group norms	3.63	0.73			.23**	.18–.29
Perceived behavioral control	3.13	1.04			.05	.00–.10

Pseudo R^2^			.17		.23	

Model 1 includes only control variables while Model 2 includes the three additional predictor variables to examine the additional variance explained above the control variables.

Standardized parameter estimates and standard error reported in table along with means (percentages) and standard deviations for study variables. A total of 2,201 participants in 92 clinic areas were used in the analysis. * p < 0.05; ** p < 0.01.

Next, we aggregated all TPB variable scores to the clinic level and identified those clinics that were in the top and bottom quartile and the middle 50% on each of the three dimensions. We then compared the mean level of participation in the ACA intervention across those groups while controlling for clinic size and teaching affiliation via ANCOVA. The overall average level of participation at the clinic level was 4.47 with a range of 2.08 to 6.46. ANCOVA results for perceived group norms were significant, F (5,76) = 5.10, p < 0.001. Post-hoc Tukey group comparisons indicated that the bottom quartile of clinics on perceived group norms had significantly lower participation scores than any other group; the difference in participation between the highest and lowest quartile groups was 1.13, d = 0.29. Similar results were observed for attitudes about the behavior, F (5,76) = 4.70, p < 0.001, and behavioral control, F (5,76) = 4.89, p < 0.001. For both of these other TPB factors, the bottom quartile clinics also had scores that were significantly lower than the other comparison groups (d = 0.28 and 0.22 respectively; see Table 3).

**Table 3:** Clinic adjusted mean and standard deviation based on ANCOVA results.

	Attitudes about the program		Perceived Group norms		Perceived behavioral control	
	Mean	SD	Mean	SD	Mean	SD

Top 25%	5.21	.78	4.68	.89	5.11	.78
Middle 50%	4.37	.88	4.77	.93	4.51	.89
Bottom 25%	3.89	.97	3.55	.71	3.72	.95

Analysis controls for academic affiliation and number of employees in clinic.

## Discussion

The present study explored the ability of variables representing three key constructs from the theory of planned behavior to explain employee participation in a workplace program designed to improve patient access to care. We predicted that each TBP factor would be related to participation, with perceived group norms being especially important.

We found partial support for the hypothesized model. Employee perceptions that group norms favored the ACA program and those whose own attitudes toward ACA were positive demonstrated greater involvement in ACA implementation. One implication of this finding is that managers looking to facilitate employee participation in an intervention may find it effective to strengthen perceived group norms regarding the intervention. This might be achieved by extensively publicizing the implementation effort and ensuring that it is thoroughly discussed in meetings by managers and coworkers. We also found attitudes about the program to be a significant predictor. This suggests that believing the program will be useful may be an incentive to participate, consistent with research. A manager could emphasize the benefits of the program by stressing the goals of the process, and how meeting the objectives can lead to better personal and customer care outcomes. The third TPB factor, perceived behavioral control, was not significant in the overall model; employee perceptions of the degree of control they had over the program (e.g, the extent to which they could adapt ACA to best suit their clinic conditions) was not a relevant factor in overall participation in this study.

With regard to the demographic and other factors included in our model, we found that level of supervisory responsibility had a large effect on participation. This is consistent with the findings and theoretical research suggesting management plays a key role implementing change programs [5,27]. The effect appeared to increase as level of responsibility increased. We also note the possibility that perceived group norms may be strengthened by participation of senior leaders in a clinic area.

In addition, employees who have been in the clinic for at least a year were more likely to participate in the program, possibly reflecting their greater identification with the clinic aims and program implementation goals. Significant effects were also found for occupation level, with nurse practitioners, registered nurses, and physicians participating in a greater number of activities compared to other clinical employees.

Contrary to expectation based on previous research, we did not find job satisfaction to be a significant predictor of participation in the ACA intervention. To explore this further, we subsequently tested a hierarchical linear mixed model that included interaction terms between job attitudes and the three key TPB predictors. None of the interaction terms were significant. These findings suggest that employee job satisfaction may not be a key factor in predicting participation in an intervention; perhaps those who are less satisfied are more motivated to implement changes that may improve their work life.

In the clinic-level analyses, we found that the level of participation was higher within clinics in the top quartile on either attitudes about the behavior or perceived behavioral control than it was in those clinics in the middle half or bottom quartile of the TPB variable distribution This suggests a linear effect for these TPB variables at a clinic level. For perceived group norms, the difference between the top quartile and middle half was not significantly different. However, the top quartile and middle clinics were both significantly higher compared to the bottom quartile clinics. We take this finding to suggest that a moderate level of peer support for an intervention may serve as a tipping point for encouraging broader employee participation in a change effort. Interventions at a clinic or group level designed to increase perceived group norms for participating in the program may lead to greater employee involvement in the proposed implementation program.

## Limitations

The study was a cross-sectional field research study and shares several of the limitations of this design. A concern is a lack of experimental control, so any causal inferences need to be examined with caution. However, there is growing sentiment that experimental controls may not always be appropriate to evaluate implementation studies. Measures for all variables were obtained the data from the same source and consequently their inter-relationships may be due in part to common method variance. However, questions about individual participation on each of the ten activities were scored dichotomously, as were demographic questions. Employees rated job satisfaction and the three components of the theory on a Likert-type scale. These differences in response options may partially mitigate the common method variance effect.

A total of 40% of the sample responded to the survey, raising concerns of non-response bias. For example, respondents may over-represent those who participated in the ACA program. We did, however, find 12% of the survey respondents indicated that no participation in any of the ACA practices. Thus, some individuals who did not participate in ACA nonetheless did complete the survey, suggesting that the present results are not based solely on the sub-sample of participants who might not be representative in some way.

## Future research

The present strong results regarding the impact of perceived group norms raises the question of whether this finding would generalize to specialty clinic areas using the same program as well as outpatient clinics trying to implement different types of clinical design programs or other innovations, such as electronic health record systems. We did not find job satisfaction to be related to participation as would be expected. Future research should examine if job satisfaction is related to other implementation activities, or if the effects of perceived group norms, attitudes, and behavioral control are more important variables and worth additional study.

We found variables based around concepts of TPB to be important predictors of participation. We would advocate a more complete test of the theory in the future as it relates to implementation programs in a team setting. Questions on the components to fully assess the sub-components and beliefs underlying each of the three predictors were used in our study as proxy measures for the constructs. Most research has examined only individual level aspects of TPB, but looking at the theory in the work setting may also lead to interesting findings and extension of prior findings. The setting and type of intervention could also be studied. The present study focused on clinical operations, but it is possible that different types of change programs may be less influenced by perceived group norms and more influenced by attitudes or perceived behavioral control.

## Summary

Our findings indicate that perceived group norms and employee's attitudes regarding the value of the intervention were related to participation in the implementation of an innovation; however, perceived behavioral control was not found to be a significant predictor. These findings partially supported hypotheses derived from the theory of planned behavior. Supervisory level also played a role in the participation. Individuals with greater managerial authority appeared to be more involved in the program. The implication would suggest that a climate where all team members recognize their colleagues are participating in the program, discussing the program at meetings, and having visible team leaders may enhance participation in operational change programs.

## Competing interests

The authors declare that they have no competing interests.

## Authors' contributions

DCM conceived this manuscript, and led the writing and analyses. CVL and MM participated in the conceptualization and drafting of this manuscript. All authors read and approved the final manuscript.
